# Applying the FAO surveillance evaluation tool (SET) to assess the fish farming disease surveillance system in Spain

**DOI:** 10.3389/fvets.2024.1399040

**Published:** 2024-07-17

**Authors:** Ana Muniesa, Imanol Ruiz-Zarzuela, Gael Lamielle, Sophie Von Dobschuetz, Dolors Furones, Chris Rodgers, Bernardo Basurco

**Affiliations:** ^1^Faculty of Veterinary Medicine, Instituto Agroalimentario de Aragón IA2 (Universidad de Zaragoza – CITA), Zaragoza, Spain; ^2^Food and Agriculture Organization of the United Nations (FAO), Rome, Italy; ^3^IRTA, La Ràpita, Tarragona, Spain; ^4^Consultant, Tarragona, Spain; ^5^Mediterranean Agronomic Institute of Zaragoza (CIHEAM-IAMZ), Zaragoza, Spain

**Keywords:** health management, surveillance evaluation tool, fish diseases, data collection, mortality, aquaculture, emerging fish pathogens

## Abstract

EU Member States should ensure that they implement adequate health surveillance schemes in all aquaculture farming areas, as appropriate for the type of production. This study presents the results of applying the FAO’s Surveillance Evaluation Tool (SET) to assess the Spanish disease surveillance system for farmed fish species, which although applied previously in livestock production, is applied here to aquaculture for the first time. Overall, there were important score differences between trout and marine fish (seabass and seabream) surveillance, which were higher for trout in the following areas: Institutional (70.8% versus 50.0%), Laboratory (91.7% versus 47.2%), and Surveillance activities (75.3% versus 61.3%). For other categories, the values were lower and no significant differences were found. However, most surveillance efforts focused only on trout, for which there are EU and WOAH listed (notifiable) diseases. In contrast, for seabream and seabass, for which there are no listed diseases, it was considered that surveillance efforts should, nevertheless, be in place and should focus on the identification of abnormal mortalities and emerging diseases, for which there are as yet no standardized harmonised methodologies.

## 1 Introduction

Aquaculture is one of the fastest growing food producing sectors in the world and is an increasingly important contributor to global food supply and economic growth. Its development is sustained by the correct biological management of the cultured species, the introduction of technological innovations in the production process, the development of new feeds and feeding systems, and the correct prevention and control of diseases (1).

Aquaculture production (fish and shellfish) in the European Union reached 1.2 million tonnes (t) in 2018 and accounted for €3.9 billion in 2020, which represented 1% of world aquaculture production by volume. The EU production is led by Spain (24%), followed by France (21%), Greece (11%), and Italy (10%) (2).

Spanish fish farming has a diversified and internationalized aquaculture sector (Table 1), both in species and production systems, with a total of 73,370 t produced in 2022 (59,123 t of marine fish, 14,122 t of freshwater fish, and 125 t of fish for restocking) (3, 6).

**Table 1:** Spanish finfish production in 2022 (3) and its relationship with notifiable diseases, according to EU and Spanish legislation, as well as the WOAH Aquatic Manual (4) and Code 2022 (5).

	Main fish species produced accounting for 98% of the total finfish production (62,900 t)							Other fish
	Rainbow trout (*O. mykiss*)	European seabass (*D. labrax*)	Gilthead seabream (*S. aurata*)	Turbot (*S. maximus*)	Bluefin tuna (*Th. thynnus*)	Meagre (*A. regius*)	Sole (*Solea* spp.)	Mullet, eel, brown trout, tench, sturgeon, Atlantic salmon
Fish production (t)	13,413	21,179	6,079	7,504	8,482	4,833	613	604
Seed production (million): trout eggs, marine fish fry	126.5	75.6	31.1	13.7	–	5.2	1.9	7.7
**Diseases listed by the European Commission implementing Regulation (EU) 2018/1882 and Spanish legislation Royal Decree 1614/2008 (listed as non-exotic diseases)**								
Viral haemorrhagic septicaemia (VHS)	EU-SL* WOAH*	EU-SL**	EU-SL**	EU-SL* WOAH*	EU-SL**	EU-SL**	EU-SL** WOAH*	EU-SL* (brown trout) EU-SL** (Mullet, tench, sturgeon) WOAH* (Atlantic salmon, brown trout)
Infectious haematopoietic necrosis (IHN)	EU-SL* WOAH*	–	–	–	–	–	–	EU-SL* (Atlantic salmon) EU-SL** (Tench, sturgeon) WOAH* (Brown trout)
Infectious salmon anaemia (ISA)	EU-SL* WOAH*	–	–	–	–	–	–	EU-SL* and OIE-Ma* (Atlantic salmon, brown trout)
**Diseases listed by the European Commission implementing Regulation (EU) 2018/1882 and in Spanish legislation Real Decreto 1614/2008 (listed as exotic diseases)**								
Epizootic haematopoietic necrosis (EHN)	EU-SL* WOAH*	–	–	–	–	–	–	EU-SL** (Tench)
**Other diseases listed in the WOAH Aquatic Manual or Code (2022) and by the European Commission implementing Decision (EU) 2021/260**								
*Gyrodactylus salaris*	WOAH*	–	–	–	–	–	–	EU* (Brown trout) WOAH* (Atlantic salmon)
Salmonid alphavirus (SAV)	EU* WOAH*	–	–	–	–	–	–	EU* and OIE-Ma* (Atlantic salmon)
Renibacteriosis	EU*	–	–	–	–	–	–	EU* (Atlantic salmon, brown trout)
Infectious pancreatic necrosis (IPN)	EU*	–	–	–	–	–	–	EU* (Brown trout)
Epizootic ulcerative syndrome (EUS)	WOAH*	–	–	–	–	–	–	WOAH* (Mullet)
Red Sea bream iridovirus (RSIV)	–	–	–	–	WOAH *	–	–	WOAH* (Mullet)
Spring viraemia of carp (SVC)	–	–	–	–	–	–	–	EU* (Tench)

EU, Diseases listed in the European regulation; EU-SL, Diseases listed in European and Spanish Legislation; WOAH, Diseases included in the WOAH Aquatic Manual and/or Code (2022); *Susceptible host species; **Vector species; t, tonnes.

As in other European countries, diseases have impacted the Spanish fish farming sector, affecting the performance and competitiveness of its industry (7, 8). Diseases may cause both a direct impact on the economic performance of a company through increasing losses (mortality, treatments, etc.) and reducing incomes, as well as having an indirect impact by affecting international trade, investments and consumer confidence (9, 10). Thus, national and international aquatic animal health programs are essential to assure the sustainable development of aquaculture, as well as to protect both the industry and the aquatic environment biodiversity of the from the negative impacts of exotic, endemic and emerging disease epizootics (11). However, all too often, a long time elapses from the first observation of an abnormal mortality in the field, to the identification and reporting of the causative agent and, even longer, to the application of appropriate control and risk management measures (12). Therefore, health management, disease surveillance and biosecurity must be part of a strategic and integrated approach that encompasses the policy and regulatory framework for analysing and managing risks to the life and health of people, animals and plants, as well as the associated risks to the environment (11).

Originally, in Europe the Council Directive 91/67/EEC of 28 January 1991 set controls mainly for salmonid (salmon, trout) and bivalve farming. Subsequently, several additional fish species, including some marine species, were listed in the Council Directive 2006/88/EC which enforced the obligation of notifying increases in mortality in aquaculture animals for further investigations. Moreover, EU Member States had to ensure that a risk-based animal health surveillance scheme was applied to all farms and mollusc farming areas, as appropriate for their type of production. Consequently, in Spain, Royal Decree 1614/2008 [implementing Directive 2006/88/CEE; (13)] provided definitions for “emerging disease” and “increase in mortality”. However, these terms can lead to different interpretations by producers and competent authorities, which in Spain are organized by subnational administrations called Autonomous Communities (AC). Nonetheless, Spain was a pioneer in structuring a surveillance system for aquaculture, both for fish farming and bivalve production, based on the risk assessment of the farms and production zones in order to prevent, not only, the occurrence, but also, the spread of the more relevant non-listed aquatic animal diseases. Moreover, the correct diagnostic methodology for use by all Spanish AC was also recommended in a guide (14). Subsequently, Directive 2006/88/CEE has been repealed by Regulation (EU) 2018/1882, which finally considers emergent diseases notifiable, when they fulfil the criteria established in Animal Health Law: AHL Regulation (EU) 2016/429 on transmissible animal diseases. The AHL aims to prevent and control all transmittable diseases in the Union. This transversal AHL enforces health surveillance and risk assessment on the operators to detect the early presence of both notifiable and emergent diseases on a fish farm. It also highlights the importance of implementing biosecurity measures to prevent the introduction of pathogens into farms.

Since disease surveillance focuses mainly on listed (notifiable) diseases both in the European Regulation EU 2020/689 (Annex VI) and at the international level, through the WOAH Aquatic Animal Health Code, the surveillance of those diseases is well-defined. However, countries also have the obligation to report emerging diseases (i.e., a new occurrence of a disease causing a significant impact); for which the indicators for defining such cases are not as well defined. Likewise, the definition of “increased mortality” leaves room for different interpretation by stakeholders, since the European legislation defines this term as “unexplained mortalities significantly above the level of what is considered to be normal for the fish farm or mollusc farming area in question under the prevailing conditions.” Therefore, what is considered as increased mortality should be objectively defined and accepted by the stakeholders responsible for production and health.

Surveillance programs aim to prevent diseases from spreading before they can affect the aquaculture industry. They can also be implemented to contain or eradicate important endemic diseases in the long term. An early detection system is an efficient system for ensuring the rapid recognition of signs that point to a suspected listed disease, or an emerging disease situation, or unexplained mortality in aquatic animals (15).

As aquaculture evolves, growing in volume, economic weight and technological development, it integrates epidemiological methodologies previously applied in terrestrial livestock/animal production systems. This is the case of the tools used for the evaluation of health surveillance systems. For example, the WOAH Tool for the Evaluation of Performance of Veterinary Services (WOAH PVS Tool), designed in 2007 and applied to livestock (16), was adapted in 2013 for aquaculture (WOAH PVS Tool: Aquatic animals) (17). Likewise, SET was designed to provide countries with a comprehensive, oriented and standardized method in order to evaluate animal disease surveillance systems, including zoonoses (18). The basis for SET was the OASIS toolkit (19), to which components of FAO’s Epidemiology and Laboratory Mapping Tools (20) were also added. More recently, a new methodology for the surveillance of diseases of aquatic organisms in developing countries has been proposed by Bondad-Reantaso et al. (9).

Several tools have been developed to help responsible health authorities and experts to evaluate and improve their surveillance programs. This is the case of the Tool for the Evaluation of Performance of Veterinary Services (WOAH PVS Tool), designed by the WOAH (previously OIE) in 2007 for livestock, which was adapted subsequently in 2013 for aquaculture (WOAH PVS Tool: Aquatic). Following a request from member countries in Africa, the FAO’s Animal Production and Health Division (NSAH) developed a surveillance evaluation tool (SET) to provide countries with a comprehensive and standardized way to evaluate animal disease surveillance systems, including zoonoses (18). The basis for the SET was the OASIS toolkit (Outil d’Analyse des Systèmes de Surveillance) from the French agency ANSES [Agence Nationale de Sécurité Sanitaire de l’Alimentation, de l’Environnement et du Travail; (19)] to which components of FAO’s Epidemiology and Laboratory Mapping Tools were added.

SET was developed by FAO in 2017 following a request from projects in African countries for an assessment tool dedicated to animal health surveillance, which could support veterinary services in developing their national surveillance systems. To date, although SET has been used in more than 25 countries in Africa and Asia for a comprehensive assessment of animal health surveillance systems. Although SET tool has not yet been applied in aquaculture, the basic concepts of animal disease surveillance are transferable to aquaculture.

SET originally consisted of a scoring grid that assessed surveillance systems against 90 indicators grouped into 19 categories and 7 major areas (Table 2). Following a thorough document review and interviews with stakeholders at all levels of a specific sector, SET scores are attributed to each indicator in order to provide information concerning the strengths and weaknesses of surveillance in any particular country. These results are then used to develop recommendations and/or a plan to implement specific, measurable and timely actions in order to track diseases better. Governments and development partners have used SET evaluations as a basis for implementing targeted improvement for animal disease surveillance systems at a national level.

**Table 2:** Areas and categories evaluated by SET.

Area	Category	No of indicators
Institutional organization	Central institutional organization	7
	Field institutional organization	8
	Intersectoral collaborations	4
Laboratory	Operational aspects	2
	Technical aspects	8
	Analytical aspects	3
Surveillance activities	Objectives and context of surveillance	4
	Surveillance data collection	14
	Surveillance procedures	9
	Animal health investigations	2
	Risk assessment	2
Epidemiology workforce	Workforce management	5
	Training	4
Data management	Information system	2
	Data processing and exploiting	5
Communications	Internal communication	4
	External communication	3
Evaluation	Internal evaluation	2
	External evaluation	2

Although there are recent studies concerning the health management and biosecurity procedures in marine fish farming in Spain (21), to our knowledge, there are no public studies concerning the performance of aquaculture surveillances system itself. The FAO SET was selected to conduct an assessment on the disease surveillance system of the Spanish fish farming sector. For that purpose, a parallel evaluation of the surveillance systems for the main fish productions of trout (freshwater), and seabream and seabass (marine fish), was conducted.

## 2 Materials and methods

This current work was implemented as a case study in order to assess the disease surveillance system for fish farming in Spain, using the FAO SET tool (18). SET is an easy, user-friendly tool, based on Excel, where most of the effort focuses on expert opinion discussions in order to score specific indicators. It requires a preliminary in depth understanding of the surveillance framework under analysis at the different levels (e.g., national, subnational, field, laboratory, communication, evaluation), which is achieved through literature and reports compilation and interviews with relevant stakeholders.

SET consists of a Microsoft Excel tool containing 90 indicators (Annex 2) covering all aspects of surveillance, which are grouped into 19 categories and 7 areas (Table 2). Following a thorough document review, as well as interviews with all relevant surveillance stakeholders, evaluators scored each SET indicator based on the realities of the surveillance system assessed. The tool then generated graphical outputs automatically, which allowed for the development of a system-specific action plan for improvement of surveillance. It is worth noting that following the use of SET in this paper, the tool has been updated by increasing the number of indicators to 96, though the tool’s categories and areas remain unchanged.

The SET evaluation consisted of six main phases:

1. *Training and preparation for SET implementation.* The full FAO SET package was shared with the Spanish team, who through several meetings familiarised themselves with the toolkit and were tutored on how interviews should be best performed. This was the first time that SET was used to evaluate aquaculture surveillance systems; therefore, it was discussed in depth and potential idiosyncrasies were addressed. The original tool developed for terrestrial animals was implemented, with the only exception being the indicator concerning the “surveillance of vectors,” which was adapted for aquaculture and changed to evaluate the “surveillance of reservoirs” instead.
2. *Review of reference documents.* A thorough search and review of international (European Union and WOAH), national (Spanish central administration) and subnational (AC) legislation, as well as operating procedures (SOPs) from the Aquaculture Health Defence Groups (ADS by their Spanish name), protocols and other written documents describing how the surveillance systems functioned was undertaken. The list of documents reviewed is shown in Annex 1.
3. *Identification of stakeholders.* Aquaculture health management and disease surveillance were carried out by a broad range of stakeholders (e.g., veterinarians and health managers, health associations, diagnostic laboratories), as well as different administrations (national and subnational). Representative stakeholders and officers engaged in the steps of fish farming disease surveillance were identified, and these included: the Ministry of Agriculture, Fisheries and Food (MAPA), through the Subdirectorate General for Animal Health and Hygiene and Traceability, the national fish disease reference laboratory, subnational administrations (AC), ADS veterinary services, and field health operators.
4. *Stakeholder interviews*. The selected stakeholders were informed about the purpose of the study and the methodology to be used. Interviews were designed to address and discuss the SET indicators with stakeholders related to their work on surveillance. Questions were sent in advance, and subsequent interviews were held face to face when possible, or alternatively by phone or internet. Most stakeholders were interviewed twice between August 2019 and April 2020. The first interviews lasted from 1 to 2 h, but the second interviews, for clarification of certain relevant indicators, were shorter.
5. *Scoring sessions.* A core evaluation team of three members was established to enter the information gathered during interviews into the SET scoring grid (Excel file). The evaluation was carried out through a series of face-to-face discussion sessions. The SET analyses for the surveillance systems were performed through the assessment of the 90 surveillance indicators, which were split into 19 categories and seven areas. Hence, for each of the 90 evaluated indicators, a score (1–4, or not applicable) was assigned together with a justification for the response. A final review of the indicators was undertaken and, if needed, the stakeholders were re-contacted for clarification. Once the scores had been input, the SET scoring grid, and graphs highlighting the surveillance system’s strengths and weaknesses were automatically generated.
6. *Development of conclusions and recommendations*. The SET scoring grids were the basis from which conclusions and recommendations were elaborated, following a descriptive analysis of the main outputs.

## 3 Results

### 3.1 Description of surveillance framework

Until recently (April 2021), the main European laws on aquatic health were Council Directive 2006/88/EC and its modification by Commission Directive 2008/53/EC amending Annex IV to Council Directive 2006/88/EC as regards spring viraemia of carp (SVC). Thus, similarly to all European Union countries, the national laws that apply to fish health management and disease surveillance in Spain were based on European Directive 2006/88EC, (recently repealed) and transposed into Spanish legislation by Royal Decree 1614/2008 on animal health requirements for aquaculture animals and products thereof, and on the prevention and control of certain diseases in aquatic animals.

The development of the SET in this study was carried out just before the current regulation came into force (Commission Implementing Regulation (EU) 2021/620), so the exercise was based on the currently repealed legislation.

Table 1 provides an overview of the production of the main fish species cultured in Spain (3) and their relationship with notifiable diseases, according to Commission Directive 2008/53/EC, together with Regulation (EU) 2018/1882 “on the application of certain disease prevention and control rules to categories of listed diseases and establishing a list of species and groups of species posing a considerable risk for the spread of those listed diseases.”

**Figure 1:**
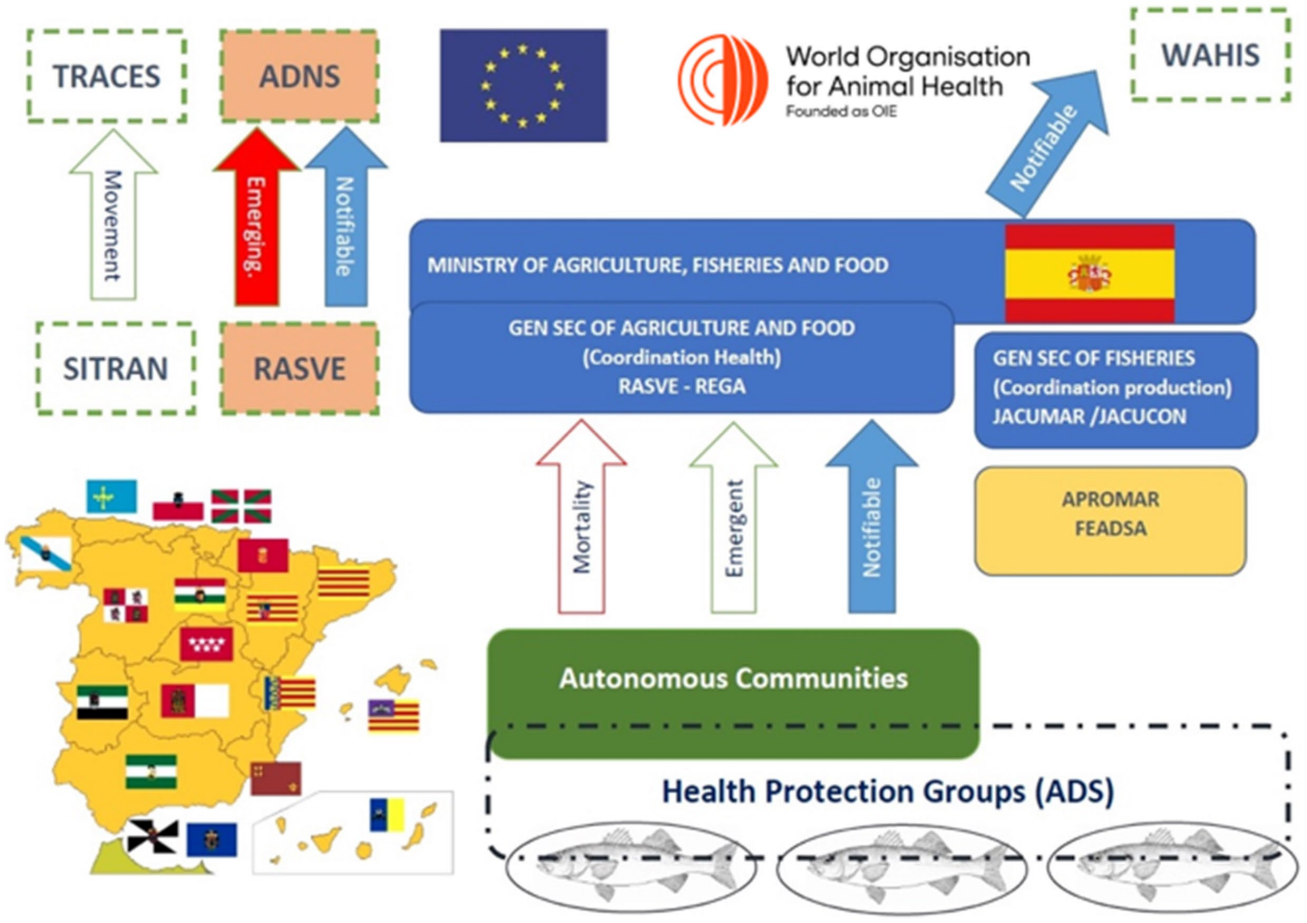
Institutional framework and fish disease reporting pathway in Spain. WAHIS (World Animal Health Information System); TRACES (Trade Control and Expert System); ADNS (Animal Diseases Notification System); SITRAN (Integrated Animal Traceability System, by its Spanish acronym); RASVE (Veterinary Health Alert Network, by its Spanish acronym).

The Spanish State is divided into 17 AC and two autonomous cities, which represent the first-order of administrative division in the country. At the national level, the executive power in Spain is exercised by the Central Government, who through its Ministry of Agriculture, Fisheries and Food, is in charge of the legal framework on animal health (i.e., transposition of European legislation into national laws and the provision of these specific laws, as well as the standards regulating the ADS), the coordination and communication with Europe and other international organizations (i.e., WOAH, FAO), and internally, coordination with the AC regarding their health surveillance systems. Therefore, as already mentioned, animal health surveillance and aquaculture are coordinated under different administrative structures in Spain, which results in the complex institutional scenario represented in Figure 1.

The AC are the responsible administrations for the implementation of the legislation on animal health and they can also develop their own additional regulation for their specific territory. Annex 1 compiles and presents the legislation, standards and guidelines that relate to fish disease surveillance in Spain at different administrative levels.

### 3.2 Description of surveillance responsibilities at the different levels

#### 3.2.1 Central administration

At the national level, the executive power in Spain is exercised by the Central Government, through the Ministry of Agriculture, Fisheries and Food, under the General Secretariat of Agriculture and Food. Within it, the responsible unit for animal health matters is the Subdirectorate General of Animal Health and Hygiene and Traceability of the General Directorate of Health of Agriculture Production.

As regards aquaculture health and surveillance, this unit (Subdirectorate General) together with the General Fisheries Secretariat coordinate the following tasks:

- Responsibility for the communication of notifiable diseases and emerging diseases to the EU, and membership of the Animal Disease Notification System (ADNS).
- Responsibility for the communication of notifiable diseases to WOAH, and membership of the World Animal Health Information System (WAHIS) (22).
- Membership of FAO, and national contacts for the Emergency Prevention System for Transboundary Animal and Plant Pests and Diseases (EMPRES).
- Responsibility for the Spanish Animal Health Surveillance Network (RASVE: Red de Alerta Sanitaria Veterinaria). RASVE collects the information from the epidemiological surveillance units of the AC and from ADNS, and relates them to the European system of the movement for animals and animal products (TRACES) and the National Traceability System (SITRAN).
- Responsibility for SITRAN, which includes the National Registry of Animal Production Units (REGA), through which farms have to report the presence of notifiable diseases.
- The central administration also has the National Reference Laboratory for Fish Diseases, based on the premises of the “Laboratorio Central de Veterinaria-LCV, Algete, Madrid,” which is responsible for the diagnosis and confirmation of outbreaks of notifiable diseases in Spain, and coordinates at the highest level with the European Reference Laboratories and, at the internal level, with the veterinary laboratories of the AC. It is also an official provider of reagents (i.e., cell lines, sera, etc.) used in diagnostics.
- Regulation of the legal action framework of the Animal Protection Groups (ADS, by their Spanish acronyms).

#### 3.2.2 Subnational administration (AC)

AC are the responsible administrations for the implementation of animal health, traceability and disease surveillance. There are 17 AC that have different administrative organizations.

All AC have an animal health unit with, among others, have duties on territorial surveys and coordination both with the national unit (through RASVE) and with the official veterinary services (i.e., field inspectors).

As well as at the national level, the functions of animal health (including aquaculture surveillance) and aquaculture production are in separate administrative structures, with the exception of the Mediterranean region of Murcia, the main producer of marine fish, where the aquaculture services are unified, and is also in charge of its own aquaculture health surveillance.

Although the AC’s have Animal Health Laboratories, few of them have expertise in fish diseases, having to rely on the National Reference Laboratory for notifiable disease for results confirmation or even routine screening. Agreements with specialised private laboratories are also complementary or alternatives for covering the gap of aquatic health diagnostic capacities at the regional level.

In addition, ACs coordinate the following tasks:

- Matching information with the ADS’s, even delegating the active and passive surveillance systems on them.
- Responsibility for informing the Central Administration (through RASVE) of notifiable and emerging diseases, plus on abnormal mortalities.
- Remit for characterizing the level of risk for fish farms and call on them according to their risk level.
- Control of farm record books (e.g., census, feed, escapees, medicines, notifiable diseases, mortalities, traceability).

#### 3.2.3 Health protection groups (ADS)

ADS are private animal health associations, operating at a Subnational level (AC), that act as the veterinary services for the sector (companies). The first ADS were created during the early 1990s for implementing health programs in trout in certain regions willing to become officially VHS- and IHN-free (23). However, currently, with the decrease of trout production, some of these ADS are not now active, whereas new ADS have been established for supervising marine fish farming in most coastal regions (Murcia, Valencia, Andalucía, Canary Islands). It is worth noting that not all farms are obliged to belong to an ADS. For instance, in Murcia and the Canary Islands all farms are members of their local ADS, whereas in Valencia they are not.

ADS are supervised by the central administration and can even receive public funds to perform certain tasks. In addition, they are responsible for the following tasks:

- Developing and implementing a health program, which includes periodic inspections, diagnosis, treatment, and epidemiological surveillance of both individual farms and production zones.
- Fish sampling, both routinely and under outbreak scenarios.
- Acting as advisors and supervisors of the farmers for their health management procedures.
- Conducting training for farmers and health managers.

### 3.3 Surveillance system evaluations

A parallel evaluation of the fish surveillance systems for trout, and seabream and seabass was performed. It was based on the analysis of information (legislation, reports, publications, etc.) and interviews with relevant stakeholders at all levels of the surveillance system (i.e., subnational, ADS, farm veterinary services). A score was given by the evaluation team, together with a justification for each of the 90 indicators in order to reflect the system’s performance for each indicator. Each one was scored from 1 (minimum) to 4 (maximum) following defined evaluation criteria. Table 2 shows the number of indicators scored by applying SET. From among the twelve indicators that resulted in a score difference of two or more, the following six stood out: “Application of quality assurance for the tests undertaken”; “Technical level of data management at the laboratory”; “Standardization of data collected”; “Acceptability of the consequences of a suspicion or case for the source or collection of data”; “Surveillance of priority diseases in susceptible wild animals”; and “Implementation of animal health investigations.”

Finally, all 90 scores were expressed as percentages based on an ideal situation where full scores were given to all indicators (100%). The system scores automatically generated graphical outputs, which thus provides a quickly performance evaluation.

Two types of outputs were generated for the surveillance system evaluations (Core Competences and Performance Attributes):

#### 3.3.1 Core competencies

**Figure 2:**
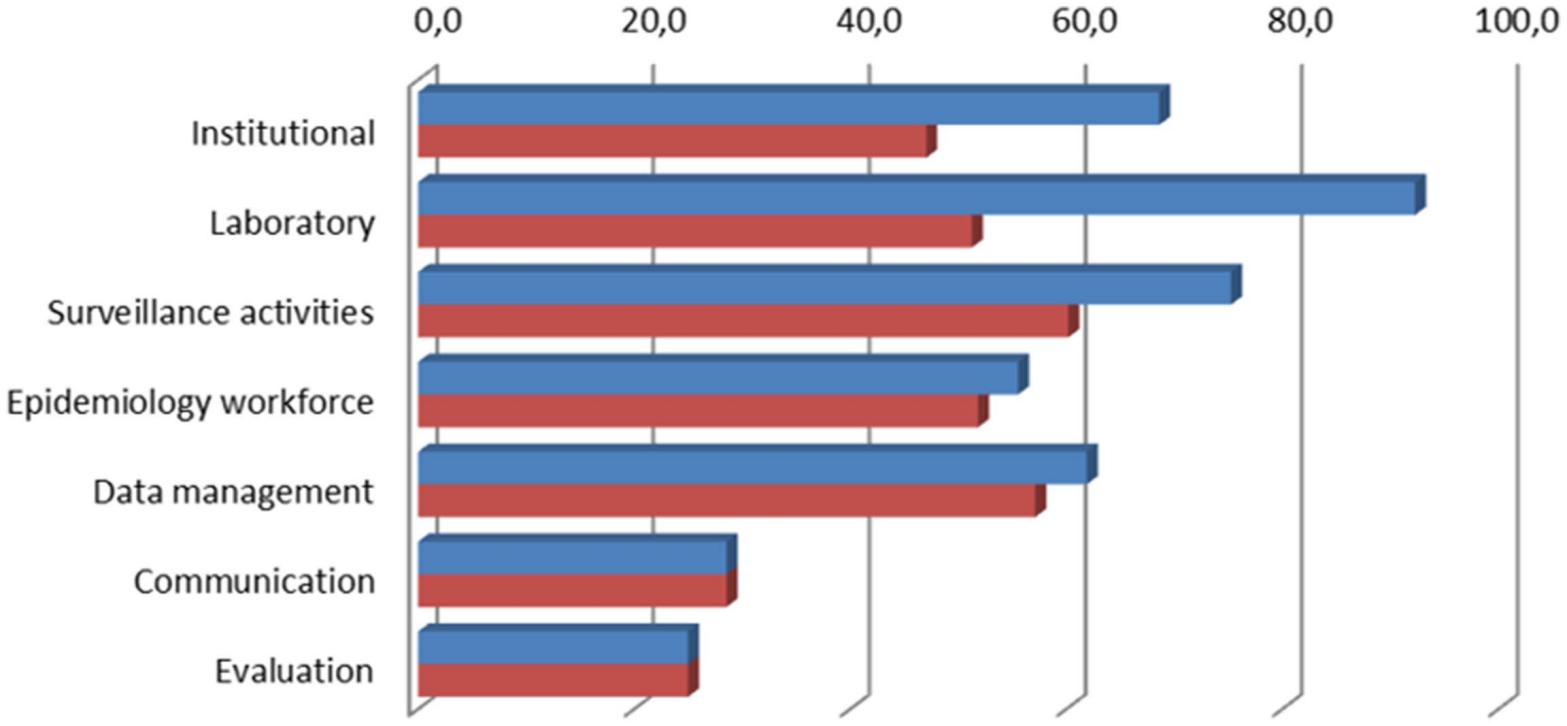
Assessment of surveillance system per SET areas. Blue bar: Trout; Red bar: Seabass and seabream.

The core results described the operational and general status of the surveillance systems, assigning a score to subcategories within each of the seven areas evaluated by the SET (Figure 2).

Overall, there were important score differences between trout and marine fish (seabass and seabream) surveillance, which were higher for trout in the following areas: Institutional (70.8% versus 50.0%); Laboratory (91.7% versus 47.2%) and Surveillance Activities (75.3% versus 61.3).

For the other categories, the values were in general lower and no significant differences between trout and marine fish (seabass and seabream) surveillance were found. Epidemiological Workforce (55.6% versus 51.9%) and Data Management (61.9% versus 57.1%) scored above 50%, and the other categories scored below 30% (Communication with 28.6% and Evaluation with 25.0%).

**Figure 3:**
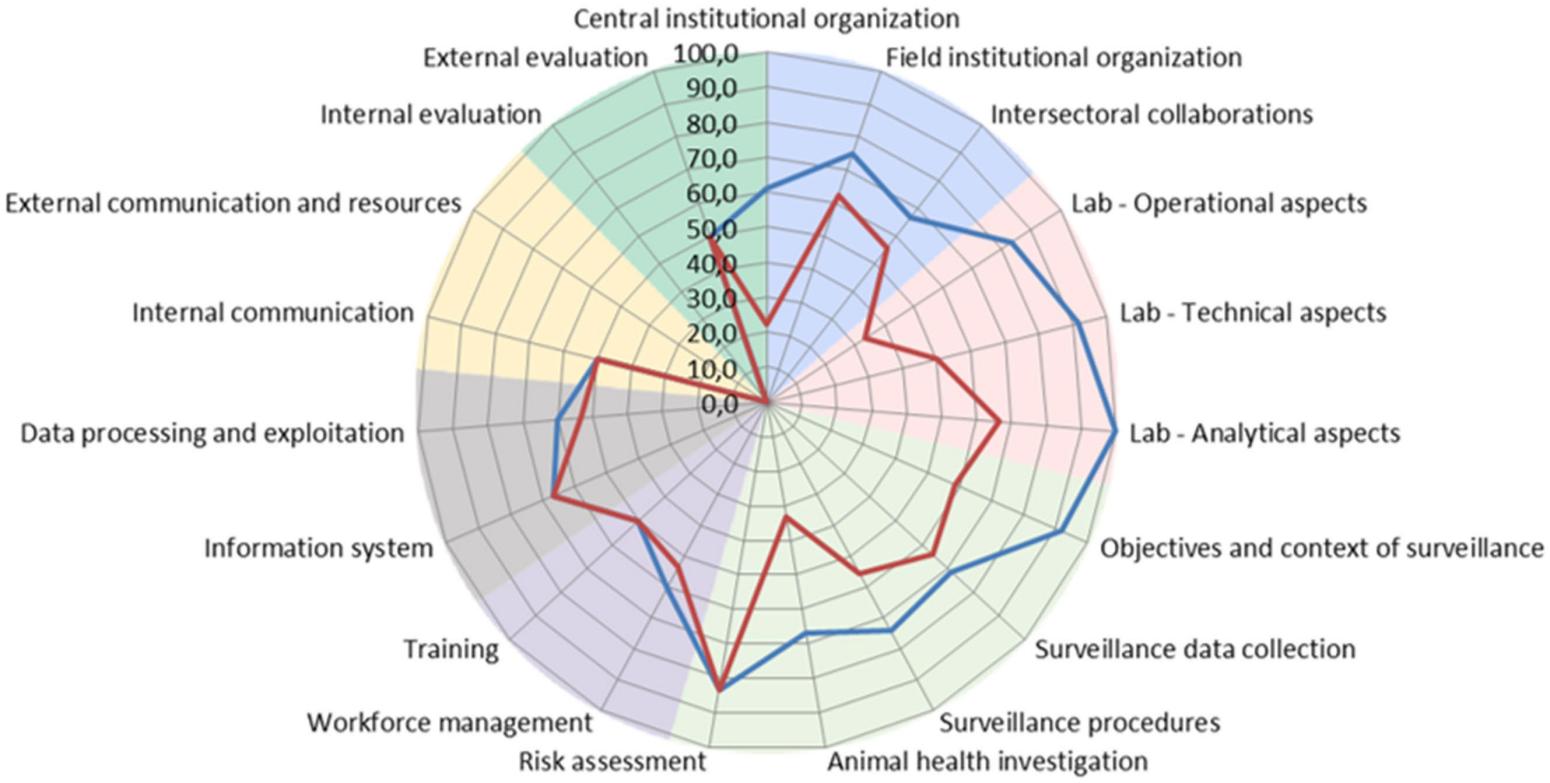
Spider graph calculated using SET comparing all results. Blue line: Trout; Red line: Seabass and seabream.

A breakdown into 19 specific categories highlighted what should be prioritized for improvement of the surveillance systems (Figure 3). The scoring outputs revealed the lowest capacity for External Communication and Internal Evaluation in both trout and marine fish (seabass and seabream). The highest score was obtained for trout in Laboratory Analytical aspects (100), Laboratory Technical aspects (91.7), and the Objectives and Context of Surveillance (91.7).

#### 3.3.2 Performance attributes

**Figure 4:**
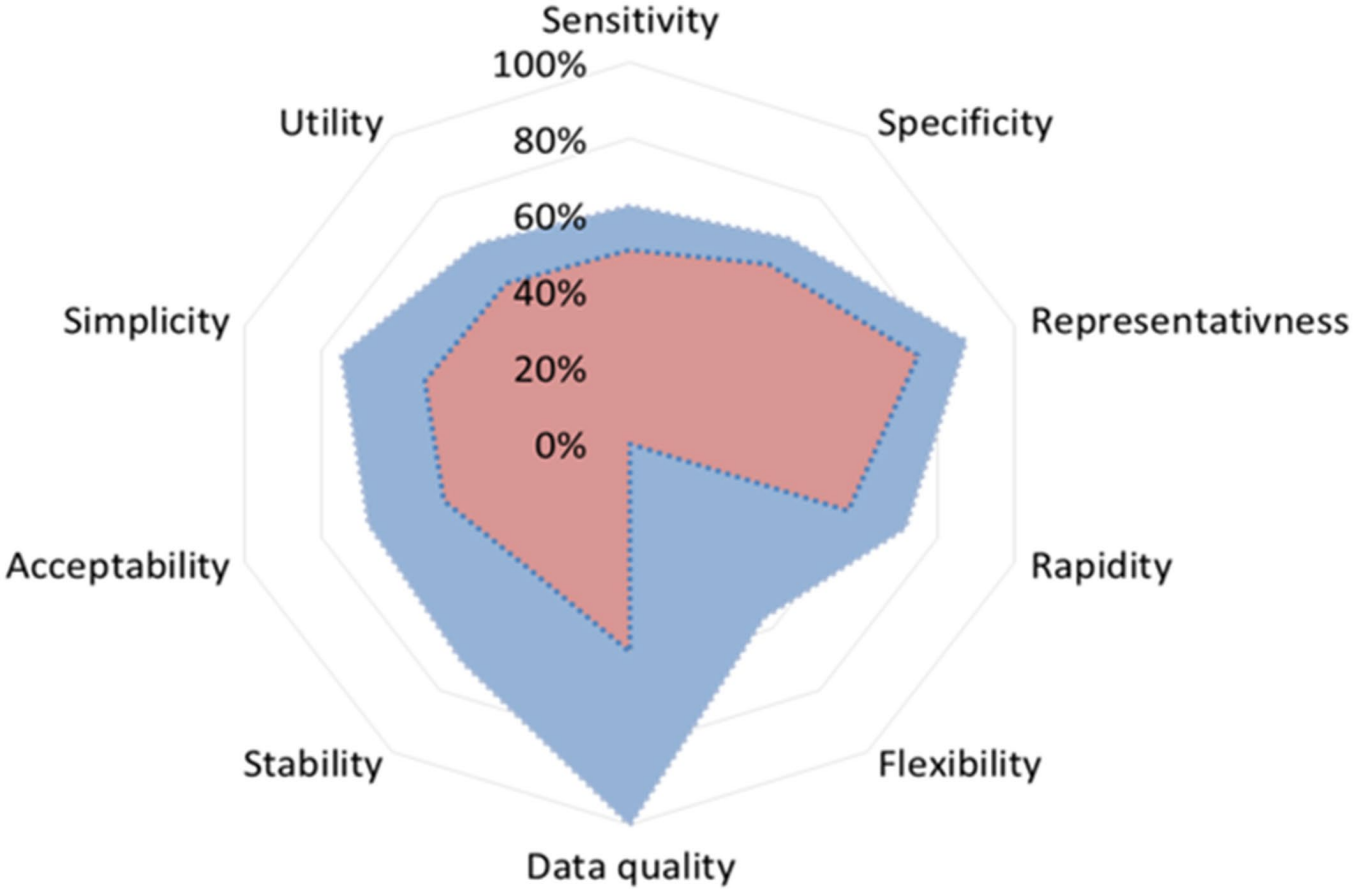
Spider graph calculated using the SET tool comparing results for performance attributes. Blue: Trout; Red: Seabass and seabream.

Performance outputs for fish disease surveillance in Spain revealed important differences in the comparison of trout and marine fish (seabass and seabream) surveillance (Figure 4), with lower scores for the second group.

Higher values were obtained for “Data quality” (100%) and “Representativeness” (88%) attributes for trout, whereas lower values were obtained for “Sensitivity” (51%) and “Acceptability” (48%) for marine fish.

## 4 Discussion

SET was shown to be easily applicable in the evaluation of surveillance in fish farming in this Spanish case study. Out of the 90 indicators in the tool, only that related to “Vector-Borne Diseases” was found not to be appropriate, and it was exchanged for a new indicator concerning “Reservoirs.”

SET is normally used to make a first evaluation with the purpose of proposing an action plan with recommendations and targeted measures to improve corresponding surveillance system. Thereafter, following this first evaluation, it is proposed to conduct follow-up evaluations every 3–5 years in order to assess the progress and improvements of the system studied. In this case, SET was used to compare the surveillance systems of trout versus marine fish (seabass and seabass), thus, providing a novel way to support the structured analysis concerning the benefits and disadvantages of surveillance programs designed for listed diseases (trout farming) versus those based only on the surveillance of abnormal mortalities and emerging diseases. Table 1 shows finfish production in Spain, the correlation between species produced and the listed (notifiable) diseases, according to the European legislation and the WOAH Aquatic Code. Thus, when analysing the correlation of species with a list of notifiable diseases, it was apparent that most of the surveillance efforts only focused on one species (trout), which accounts for less than one sixth of the total finfish production in Spain. In contrast, for seabream and seabass, for which there are no listed diseases, surveillance efforts should be in place in order to focus on the identification of abnormal mortalities and emerging diseases.

SET provided the highest scores for trout surveillance in Laboratory Technical and Laboratory Analytical aspects. This is because the Spanish reference laboratory for fish diseases is only obliged to devote human and technical resources towards diagnosing listed diseases. Another important point is that the surveillance systems are always alert to the possible occurrence of a listed disease. For example, a suspected case of viral haemorrhagic septicaemia (VHS) based on high mortality and compatible clinical signs (which are very similar to signs exhibited by other haemorrhagic diseases, such as enteric redmouth disease, furunculosis, vibriosis, and infectious salmon anaemia; WOAH Aquatic Manual, 2022), is sufficient to open an investigation by competent authorities. The official veterinary services have well-established procedures for performing surveillance on notifiable diseases, such as VHS, for which there are well-defined guidelines related to the definition of suspected cases, as well as early communication procedures, diagnosis and confirmation. However, for marine fish (seabass and seabream), an official case investigation will only be opened if abnormal mortalities are detected. Nevertheless, in this case, except for Murcia Autonomous Community, the indicator of abnormal mortality is not defined, and any sampling is undertaken by the affected farms and the corresponding ADS, as there are no official laboratories for the diagnosis of marine fish diseases, and the official veterinary services do not have well-established procedures for performing surveillance of abnormal mortalities and emerging diseases. It is worth noting though that although Spain benefits from an experienced institutional and private laboratory network, it is not closely connected to the official network and, as such, loses very valuable capacity and information input that is essential for correct management of the health system (24).

Council Directive 2006/88/EC endorsed Member States to approve national measures for limiting the impact of certain relevant diseases in aquaculture and wild aquatic animals not listed in Part II of Annex IV of that Directive. Consequently, the Commission Decision of 15 April 2010 approved national measures and eradication programs regarding certain diseases [i.e., spring viraemia of carp (SVC), bacterial kidney disease (BKD), infectious pancreatic necrosis virus (IPN) and infection with *Gyrodactylus salaris* (GS)] in Denmark, Finland, Ireland, Sweden, and United Kingdom. However, no measures/eradication programs were approved for any Mediterranean country, despite the growing economic magnitude of the marine fish farming sector and the known impact of certain diseases, such as the case of nodavirus (7, 25, 26).

The two lowest rated categories in SET for both trout and marine fish (seabass and seabream) were “External Communication and Resources” and “Internal Evaluation.” This showed that there was limited and/or inefficient exchange of information on health and disease aspects between the competent authorities and the stakeholders involved (e.g., administration, producers, laboratories, pharmaceutical companies, insurance companies, research centres and universities). In this context, it is very important to value the need for information exchange, as occurs in other countries (27–29) where all cases of disease are reported in a public database, so that any interested stakeholder can consult veterinary inspection reports and mortality farm records. Additionally, competent surveillance authorities and laboratories pass external evaluation exercises, although there is a lack of internal evaluation procedures.

It should be pointed out that most animal production also takes place in inland locations, where animal health surveillance and official veterinary services are well represented. On the other hand, marine fish production takes place in environments (on the coast and off-shore) where, generally, official veterinary services are lacking in both proficiency and a clear legislative framework that can guide their actions. Another observed constraint is that terrestrial animal production units and animal health units are under the same administrative structure, whereas fish farming health and production units are under different administrative structures, both at the national and subnational levels, thus, generating additional burdens for communication and information readiness.

## 5 Conclusion

The conclusions and recommendations derived from this study could be of value not only for Spain, but also for fish health authorities and experts that would like to conduct similar assessments of fish disease surveillance systems in other countries. The FAO SET tool has been shown to be very suitable for this purpose, and showed significant higher scores for trout when compared to marine fish (seabass and seabream) surveillance. The main reasons behind this difference seemed to be based on the fact that surveillance for trout is always alert for the possible occurrence of a listed disease (e.g., VHS), whereas surveillance for marine fish (seabass and seabream) should react only after the occurrence of abnormal mortalities, for which standardized methodologies have yet to be implemented.

Therefore, in conclusion, it is recommended that the Spanish marine fish surveillance plan needs to be improved at the national level for seabass and seabream, since marine species have higher production levels and value. In order to assure that the implemented surveillance system meets its objectives, by improving quality and efficiency, it is also recommended to organize periodic re-assessments.

## Data availability statement

The original contributions presented in the study are included in the article/Supplementary material, further inquiries can be directed to the corresponding author.

## Ethics statement

The manuscript presents research on animals that do not require ethical approval for their study.

## Author contributions

AM: Data curation, Formal analysis, Investigation, Methodology, Writing – original draft, Writing – review & editing. IR-Z: Conceptualization, Methodology, Writing – original draft, Writing – review & editing. GL: Conceptualization, Formal analysis, Methodology, Writing – review & editing. SD: Validation, Writing – review & editing. DF: Supervision, Writing – review & editing. CR: Conceptualization, Supervision, Writing – review & editing. BB: Conceptualization, Formal analysis, Methodology, Writing – original draft, Writing – review & editing.
