# Supplementary material for: Applying the FAO surveillance evaluation tool (SET) to assess the fish farming disease surveillance system in Spain
Source: Front Vet Sci. 2024 Jul 17;11:1399040. doi: 10.3389/fvets.2024.1399040 (PMC11290467; doi:10.3389/fvets.2024.1399040)
Supplement: Supplementary file 1 [file Table_1.DOCX]

**Annex 2**

**Table 1. Indicators’ scores for trout and marine fish (seabass and seabream) surveillance applying the FAO Surveillance Evaluation Tool (SET).** Each indicator was scored between 1 (minimum) to 4 (maximum) following defined criteria for evaluation, analysis of information and group experts’ discussion. Trout and marine fish indicator scores with a difference of two or more are marked in orange.

| **Area** | **Category** | **Indicator** | **Trout** | **Seabass / seabream** |
| --- | --- | --- | --- | --- |
| **Institutional organization** | Central institutional organization | Existence of an operational management structure (central unit) | 3 | 2 |
|  |  | Existence of a steering committee that is representative of the partners | **3** | **1** |
|  |  | Existence of a scientific and/or technical committee for the system | 2 | 2 |
|  |  | Organization and operations of the system laid down in regulations, a formal agreement or a convention established between the partners | 3 | 2 |
|  |  | Frequency of meetings of the central coordinating body | **4** | **2** |
|  |  | Supervision of intermediary units by the central level | 2 | 2 |
|  |  | Adequacy of the central level’s material and financial resources | 2 | 1 |
|  | Field institutional organization | Existence of formal intermediary units covering the entire territory | 4 | 4 |
|  |  | Active role of intermediary units in the function of the system (data validation, management, feedback) | 3 | 2 |
|  |  | Implementation of supervision by the intermediary level | 4 | 4 |
|  |  | Harmonization of intermediary units’ activities | 3 | 2 |
|  |  | Adequacy of material and financial resources of intermediary units only (field will be addressed later on) | 3 | 2 |
|  |  | Existence of coordination meetings at the intermediary level | 3 | 3 |
|  |  | Representativeness of the field agents’ coverage of population under surveillance | 3 | 3 |
|  |  | Adequacy of material and financial resources at the field level only | 3 | 3 |
|  | Intersectoral collaborations | Coordination with private sector | 3 | 3 |
|  |  | Coordination with Public Health Sector | 3 | 3 |
|  |  | Coordination with Environmental Health Sector | 3 | 2 |
|  |  | Coordination mechanism are established on priority zoonotic diseases between MoH (Minsitry of Health) and MoA (Minsitry of Agriculture - or equivalent), WHO, FAO, OIE, etc. | N/A | N/A |
| **Laboratory** | Operational aspects | Effective integration of laboratories in the surveillance system | **4** | **2** |
|  |  | Adequacy of human, material, and financial resources for diagnostic needs conforming to the needs of the system | 3 | 2 |
|  | Technical aspects | Application of Quality Assurance for the tests undertaken | **4** | **1** |
|  |  | Level of the standardization of work between different laboratories | 4 | 3 |
|  |  | Proportion of tests for priority diseases submitted to inter-laboratory proficiency testing (samples sent for confirmation testing are not included) | 4 | 3 |
|  |  | Availability of a diagnostic investigation personnel to support field investigation agents | 2 | 2 |
|  |  | Relevance of diagnostic techniques | 4 | 2 |
|  |  | Sensitivity of diagnostic techniques for priority diseases | 4 | 4 |
|  |  | Specificity of diagnostic techniques for priority diseases | 4 | 3 |
|  |  | Control of laboratory reagents | **4** | **2** |
|  | Analytical aspects | Technical level of data management at the laboratory | **4** | **2** |
|  |  | Analysis deadlines at the laboratory between sample analysis and reporting of results to the central unit (formalization, standardization, verification, transfer of results to the central epidemiological unit) | 4 | 4 |
|  |  | Quality of laboratory reports delivering results | 4 | 3 |
| **Surveillance activities** | Objectives and context of surveillance | Relevance of surveillance objectives | 4 | 3 |
|  |  | Level of detail, accuracy, and formalization of objectives | **4** | **2** |
|  |  | Taking partners’ expectations into account | 3 | 3 |
|  |  | Consistency of the priority diseases under surveillance with the sanitary situation (existing/exotic diseases or threats) | 4 | 3 |
|  | Surveillance data collection | Existence of a formalized surveillance protocol for each disease or threat under surveillance | 4 | 3 |
|  |  | Standardization of data collected | **3** | **1** |
|  |  | Relevance of data collection tools (excluding laboratory tools) | 2 | 2 |
|  |  | Sensitivity of the case or threat definition | 3 | 3 |
|  |  | Specificity of the case or threat definition | 4 | 4 |
|  |  | Simplicity of the case or threat definition | **4** | **2** |
|  |  | Quality of the completion of investigation forms | 1 | 1 |
|  |  | Relevance of collected samples | 4 | 4 |
|  |  | Standardization of collected samples | 4 | 4 |
|  |  | Quality of samples collected | 4 | 4 |
|  |  | Defined intervals between the detection of a case or threat and the delivery of results | 3 | 3 |
|  |  | Simplicity of the notification procedure from the field to the central level | 3 | 3 |
|  |  | Simplicity of the data collection procedure | 4 | 4 |
|  |  | Acceptability of the consequences of a suspicion or case for the source or collector of data | **1** | **3** |
|  | Surveillance procedures | Appropriateness of surveillance procedures with the system's objectives | 4 | 4 |
|  |  | Existence of passive (event-based) surveillance whose results are representative | 3 | 2 |
|  |  | Existence of awareness building programs for data sources in a passive (event-based) surveillance system | 1 | 1 |
|  |  | Relevance and suitability of active (planned) surveillance protocols | 4 | 3 |
|  |  | Surveillance of priority diseases in susceptible wild animals | **3** | **1** |
|  |  | RESERVOIR surveillance and control | 2 | 1 |
|  |  | Representativeness of the populations under active (planned) surveillance (selection bias) | 4 | 4 |
|  |  | Precision of sample under active (planned) surveillance (sample size) | 4 | 4 |
|  |  | Completeness of active (planned) surveillance | 4 | 4 |
|  | Animal health investigation | Animal health investigation teams and rapid response teams | 2 | 2 |
|  |  | Implementation of animal health investigations | **4** | **2** |
|  | Risk assessment | Implementation of animal health risk assessment | 4 | 4 |
|  |  | Usefulness of risk assessment activities for informing surveillance priorities | 3 | 3 |
| **Epidemiology workforce** | Workforce management | Terms of Reference (ToRs) | 4 | 3 |
|  |  | Manpower for Epidemiology Activities | 1 | 1 |
|  |  | Human Resource Planning | 2 | 2 |
|  |  | Minimum qualifications of staff conducting epidemiological analyses | 4 | 4 |
|  |  | Adequate skill level in epidemiology of members of the central unit | 3 | 3 |
|  | Training | Initial training implemented for all field agents when joining the system | 3 | 3 |
|  |  | Objectives and contents of initial training of system field actors adequate for operational surveillance needs | 3 | 3 |
|  |  | Regular refresher training specific to the surveillance system | 2 | 2 |
|  |  | Adequacy of material and financial resources for training | 2 | 2 |
| **Data management** | Information system | Adequacy of the data management system for the needs of the system (relational database, etc.) | 2 | 2 |
|  |  | Data input interval in accordance with the objectives and use of system results | 4 | 4 |
|  | Data processing and exploiting | Designated staff available and trained in data entry, management and analysis. | 3 | 3 |
|  |  | Adequacy of material and financial resources for data management and analysis | 3 | 3 |
|  |  | Data verification and validation procedures formalized and operational | 2 | 2 |
|  |  | Complete descriptive processing of data | 3 | 3 |
|  |  | Analysis of data fits the needs of the system | 3 | 2 |
| **Communication** | Internal communication | Regular release of reports on surveillance results (including reports to international organizations such as and/or OIE) | 4 | 4 |
|  |  | Reporting of individual test results to field actors and data collectors | 3 | 3 |
|  |  | Systematic distribution of reports on results to field actors (outside of a bulletin). | 1 | 1 |
|  |  | Presence of a communications system organized horizontally and vertically between field actors (mail, web, telephone…) | 2 | 2 |
|  | External communication and resources | Regular dissemination of a relevant information newsletter | 1 | 1 |
|  |  | Solid external communication policy with decision makers and other partners (exclude mandatory reporting to AU-IBAR or OIE) | 1 | 1 |
|  |  | Adequacy of material and financial resources for communication | 1 | 1 |
| **Evaluation** | Internal evaluation | System of performance indicators developed and validated by the directors of the surveillance system | 1 | 1 |
|  |  | Performance indicators regularly measured, interpreted, and disseminated | 1 | 1 |
|  | External evaluation | External evaluations carried out | 3 | 3 |
|  |  | Implementation of corrective measures | 2 | 2 |
