# Supplementary material for: Applying the FAO surveillance evaluation tool (SET) to assess the fish farming disease surveillance system in Spain
Source: Front Vet Sci. 2024 Jul 17;11:1399040. doi: 10.3389/fvets.2024.1399040 (PMC11290467; doi:10.3389/fvets.2024.1399040)
Supplement: Supplementary file 2 [file Presentation_1.pdf]

Annex 1. Legal framework and main rules and guidelines adopted in Spain with relation to fish disease surveillance at different administrative levels.

1. WOAH

- Aquatic Animal Health Code (2022). https://www.woah.org/en/what-we-do/standards/codes-and-manuals/aquatic-code-online-access/

- Manual of Diagnostic Tests for Aquatic Animals (2022). https://www.woah.org/en/what-we-do/standards/codes-and-manuals/aquatic-manual-online-access/

2. European Union

- *Council Directive* of 28 January 1991 concerning the animal health conditions governing the placing on the market of aquaculture animals and products (91/67/EEC). https://eurlex.europa.eu/legalcontent/EN/TXT/PDF/?uri=CELEX:31991L0067&from=en

- *Regulation (EU) 2016/429* of the European Parliament and of the Council of 9 March 2016 on transmissible animal diseases and amending and repealing certain acts in the area of animal health (Animal Health Law). https://eur-lex.europa.eu/legal-content/EN/TXT/?uri=CELEX:32016R0429

- Commission Implementing *Regulation (EU) 2018/1882* of 3 December 2018 on the application of certain disease prevention and control rules to categories of listed diseases and establishing a list of species and groups of species posing a considerable risk for the spread of those listed diseases. https://eur-lex.europa.eu/eli/reg_impl/2018/1882/oj

- Commission Implementing *Regulation (EU) 2020/2002* of 7 December 2020 laying down rules for the application of Regulations (EU) 2016/429 of the European Parliament and of the Council with regard to Union notification and Union reporting of listed diseases, to formats and procedures for submission and reporting of Union surveillance programmes and of eradication programmes and for application for recognition of disease-free status, and to the computerised information system.

- Commission Implementing *Regulation (EU) 2020/2236* of 16 December 2020 laying down rules for the application of Regulations (EU) 2016/429 and (EU) 2017/625 of the European Parliament and of the Council as regards model animal health certificates for the entry into the Union and movements within the Union of

consignments of aquatic animals and of certain products of animal origin from aquatic animals, official certification regarding such certificates and repealing Regulation (EC) 1251/2008.

- Commission Delegated *Regulation (EU) 2020/689* of 17 December supplementing Regulation (EU) 2016/429 of the European Parliament and of the Council as regard rules for surveillance, eradication programmes, and disease-free status for certain listed and emerging diseases. https://eur-lex.europa.eu/legal-content/ES/TXT/PDF/?uri=CELEX:32020R0689&from=EN

- Commission Implementing *Decision (EU) 2021/260* of 11 February 2021 approving national measures designed to limit the impact of certain diseases of aquatic animals in accordance with Article 226 (3) of Regulation (EU) 2016/429 of the European Parliament and of the Council and repealing Commission Decision 2010/221/EU.

- Commission Implementing *Regulation (EU) 2021/620* of 15 April 2021 laying down rules for the application of Regulation (EU) 2016/429 of the European Parliament and of the Council as regards the approval of the disease-free and non-vaccination status of certain Member States or zones or compartments thereof as regards certain listed diseases and the approval of eradication programmes for those listed diseases.

3. Spain

- *Ley 8/2003*, de 24 de abril, de sanidad animal (BOE núm. 99, de 25 de abril de 2003).

- *Real Decreto 1440/2001*, de 21 de diciembre, por el se establece el sistema de alerta sanitaria veterinaria (BOE núm. 12, de 14 de enero de 2002).

- *Real Decreto 1614/2008*, de 3 de octubre, relativo a los requisitos zoosanitarios de los animales y de los productos de la acuicultura, así como a la prevención y el control de determinadas enfermedades de los animales acuáticos (BOE núm. 242, de 7 de octubre de 2008).

- *Real Decreto 526/2014*, de 20 de junio, por el que se establece la lista de las enfermedades de los animales de declaración obligatoria y se regula su notificación (BOE núm. 167, de 10 de julio de 2014).

- *Real Decreto 81/2015,* de 13 de febrero, por el que se establecen las bases reguladoras de las subvenciones estatales destinadas a las agrupaciones de defensa sanitaria ganaderas (BOA núm. 50, de 27 de febrero de 2015).

- MAGRAMA (Ministerio de Agricultura, Alimentación y Medio Ambiente) 2015. Manual práctico de operaciones en la lucha contra las enfermedades de los animales acuáticos. Enero de 2015. Available at:

https://www.mapa.gob.es/es/ganaderia/temas/sanidad-animal-higiene-ganadera/manual_operaciones_acuaticos_enero2015_tcm30-111447.pdf

- MAGRAMA (Ministerio de Agricultura, Alimentación y Medio Ambiente), 2015b. Plan Estratégico Plurianual de la Acuicultura Española 2014 – 2020. Documento de Planificaciones Estratégicas Autonómicas. March 2015. Available at: https://www.mapa.gob.es/es/pesca/temas/acuicultura/315_PLANES_AUTONOMICOS_PEAE_MAR_2015_tcm30-77596.pdf

- MAGRAMA (Ministerio de Agricultura, Alimentación y Medio Ambiente), 2015c. Plan Estratégico Plurianual de la Acuicultura Española 2014 – 2020. July 2015. Available at: https://www.mapa.gob.es/es/pesca/temas/acuicultura/plan_estrategico_6_julio_tcm30-77594.pdf

- MAGRAMA (Ministerio de Agricultura, Alimentación y Medio Ambiente). Sistema de vigilancia zoosanitaria basado en el riesgo en animales acuáticos. Available at: https://www.mapa.gob.es/es/ganaderia/temas/sanidad-animal-higiene-ganadera/sistema_vigilancia_zoosanitaria_riesgo_animales_acuaticos_tcm30-376888.pdf

4. Autonomous communities

- Aragón. *Decreto 138/1992*, de 7 de julio, de la Diputación General de Aragón, por el que se regulan las Agrupaciones de Defensa Sanitaria en la Comunidad Autónoma de Aragón (BOA núm. 83, de 20 de julio de 1992).

- Comunidad de Castilla y León. *Orden AYG/1138/2012*, de 14 de diciembre, por el que se regula la Base de Datos del Registro de Explotaciones Ganaderas de castilla y León (BOCYL núm. 436, de 4 de enero de 2013).

- Comunidad Valenciana. *Resolución de 24 de abril de 2019*, de la Conselleria de Agricultura, Medio Ambiente, Cambio Climático y Desarrollo Rural, por la que se convocan para el año 2019, las ayudas a la gestión sanitaria y de bienestar animal en la acuicultura de la Comunitat Valenciana. [2019/4782] (DOGV núm. 8546, de 13 de mayo de 2019).

- Comunidad Foral de Navarra. *Orden Foral 93/2017*, de 28 de febrero de la Consejera de Desarrollo Rural, Medio Ambiente y Administración Local, por la que se modifica la Orden Foral 230/2015, de 5 de junio, por la que se aprueban las normas reguladoras para la concesión de ayudas a las Agrupaciones de Defensa Sanitaria Ganaderas, por la realización de programas sanitarios de prevención, lucha y erradicación de

enfermedades de los animales, se establece el contenido mínimo de los programas sanitarios de las Agrupaciones de Defensa Sanitaria Ganaderas (BON núm. 74, de 18 de abril de 2017).

- Junta de Andalucía. Consejería de Agricultura, Pesca y Desarrollo Rural. *Orden de 18 de julio de 2018*, por la que se convocan para el periodo 2018-2019, las ayudas previstas en la Orden de 27 de octubre de 2017, por la que se aprueban las bases reguladoras para la concesión de subvenciones en régimen de concurrencia competitiva dirigidas a la ejecución de programas sanitarios en Andalucía a través de las Agrupaciones de Defensa Sanitaria Ganadera (BOJA núm. 142, de 24 de julio de 2018).

- Región de Murcia. *Decreto núm. 19/2018,* de 14 de marzo, del Consejo de Gobierno, por el que se aprueban las normas especiales reguladoras de una subvención a otorgar mediante concesión directa por la Consejería de Agua, Agricultura, Ganadería y Pesca a la Agrupación de Defensa Sanitaria del Sector Acuícola de la Región de Murcia, para la ejecución del 'Programa Sanitario Común ADS Región de Murcia (BORM núm. 64, de 17 de marzo de 2018).
